# Performance of the minimally invasive autopsy tool for cause of death determination in adult deaths from the Brazilian Amazon: an observational study

**DOI:** 10.1007/s00428-019-02602-z

**Published:** 2019-06-14

**Authors:** Antonio E. M. Palhares, Luiz Ferreira, Monique Freire, Paola Castillo, Miguel J Martínez, Juan Carlos Hurtado, Natalia Rakislova, Rosauro Varo, Mireia Navarro, Isaac Casas, Jordi Vila, Wuelton M. Monteiro, Ariadna Sanz, Llorenç Quintó, Fabiola Fernandes, Carla Carrilho, Clara Menéndez, Jaume Ordi, Quique Bassat, Marcus Lacerda

**Affiliations:** 1 Universidade do Estado do Amazonas, Manaus, Amazonas 69850-000, Brazil; 2 Fundação de Medicina Tropical Dr. Heitor Viera Dourado, Manaus 69040-000, Brazil; 3 ISGlobal-Hospital Clínic, Universitat de Barcelona, Villarroel 170, 08036 Barcelona, Spain; 4 Department of Pathology, Hospital Clinic - Universitat de Barcelona, Villarroel 170, 08036 Barcelona, Spain; 5 Department of Microbiology, Hospital Clinic - Universitat de Barcelona, 08036 Barcelona, Spain; 6 Centro de Investigação em Saúde de Manhiça (CISM), 1929 Maputo, Mozambique; 7 National Council for Scientific and Technological Development (CNPq, Brasilia, Brazil) fellow, Brasília, Brazil; 8 Department of Pathology, Maputo Central Hospital, 1653 Maputo, Mozambique; 9 Faculty of Medicine, Eduardo Mondlane University, 1653 Maputo, Mozambique; 10 ICREA, Catalan Institution for Research and Advanced Studies, Passeig de Lluís Companys 23, 08010 Barcelona, Spain; 11 Paediatric Infectious Diseases Unit, Paediatrics Department, Hospital Sant Joan de Déu - Universitat de Barcelona, 8950 Barcelona, Spain; 12 Consorcio de Investigación Biomédica en Red de Epidemiología y Salud Pública (CIBERESP), 28029 Madrid, Spain

**Keywords:** Cause of death, Minimally invasive autopsy, Complete autopsy, Infectious diseases, Clinico-pathological discrepancies

## Abstract

The uncertainty about the real burden of causes of death (CoD) is increasingly recognized by the international health community as a critical limitation for prioritizing effective public health measures. The minimally invasive autopsy (MIA) has shown to be a satisfactory substitute of the complete diagnostic autopsy (CDA), the gold standard for CoD determination in low- and middle-income countries. However, more studies are needed to confirm its adequate performance in settings with different epidemiology. In this observational study, the CoD obtained with the MIA were compared with the clinical diagnosis and the results of the CDA in 61 deaths that occurred in an infectious diseases referral hospital in Manaus, Brazilian Amazon. Concordance between the categories of diseases obtained by the three methods was evaluated by the Kappa statistic. Additionally, we evaluated discrepancies between clinical and complete diagnostic autopsy diagnoses. The MIA showed a substantial concordance with the CDA (Kappa = 0.777, 95% CI 0.608–0.946), and a perfect or almost perfect coincidence in specific diagnosis (ICD-10 code) between MIA and CDA was observed in 85% of the cases. In contrast, the clinical diagnosis showed a fair concordance with the CDA (Kappa = 0.311, 95% CI 0.071–0.552). Major clinico-pathological discrepancies were identified in 49% of cases. In conclusion, the MIA showed a substantial performance for CoD identification. Clinico-pathological discrepancies remain high and justify the need for post-mortem studies, even in referral hospitals. The MIA is a robust substitute of the CDA for CoD surveillance and quality improvement of clinical practice in low- and middle-income settings.

## Introduction

The uncertainty about the real burden of causes of death (CoD) is increasingly recognized by the international health community and the funding agencies as a critical limitation for prioritizing effective public health measures [1]. The complete diagnostic autopsy (CDA) is the gold standard to determine the CoD, but despite its benefits, the autopsy rates are declining worldwide [2–5]. The reasons are multiple and include the invasive nature of the procedure, which hampers its acceptability among next-of-kin and physicians, as well as the time spent by the pathologists and the high costs of the procedure [6, 7]. An additional reason is the perception of the clinicians that in this era of high-tech imaging exams and accurate laboratory tests, autopsies are no longer necessary [6]. However, major discrepancies between the clinical diagnosis and the final autopsy diagnosis are still relatively frequent [8, 9].

In high-income countries, imaging-based methods have been proposed as an alternative to the CDA, but the high costs and the high level of expertise required are major drawbacks for their implementation, particularly in low- and middle-income countries (LMIC). Moreover, these imaging-based methods could be insufficient for the identification of infectious CoD [10], presumably more frequent in LMIC. Therefore, innovative methods are necessary in LMIC, where most premature deaths occur, and robust mortality data are poor [1].

In recent years, the minimally invasive autopsy (MIA), a simple method based on needle sampling of fluids and key organs followed by histological and microbiological analyses, has been developed and validated in all age groups, including stillborn babies, neonates, children, and adults, including maternal deaths [11–14] in Mozambique. The concordance between the MIA and the CDA varies in the different age groups, ranging from moderate to substantial, and is particularly high for infectious diseases [11–14]. However, the MIA has only been tested in Mozambique and has not been evaluated in other LMIC with different epidemiological backgrounds. We hereby aimed to validate the MIA approach against the CDA and to assess the concordance between the clinical diagnoses and the CDA diagnoses in a series of deaths from an infectious diseases tertiary hospital in Manaus, Western Brazilian Amazon.

## Materials and methods

### Study setting and design

This observational study was conducted at the *Fundação de Medicina Tropical Dr. Heitor Vieira Dourado* (FMT-HVD), a tertiary hospital, which is a national and international referral centre for the diagnosis and treatment of tropical diseases. The Institution is located in the municipality of Manaus, in the North East of the Amazonian State. The city has an estimated population of 2,094,301 inhabitants, of whom the majority reside in urban and peri-urban areas [15]. Major imaging exams and laboratory tests are fully available at FMT-HVD.

Deaths of any age having occurred within the hospital from March 2014 to February 2015 were eligible for recruitment. All patients had been referred to the FMT-HVD with the suspicion of infectious diseases. The inclusion criteria were as follows: (1) a CDA requested by the clinician as part of the medical evaluation of the patient, (2) written informed consent to perform the autopsy given by the relatives and (3) lapse of time between death and autopsy procedure of less than 48 h.

The study received the approval of the Clinical Research Ethics Committee of the Hospital Clinic of Barcelona (File 3024/9688) and the local (Manaus, Amazonas state) and National (Brazil) Bioethics C omm itt ees ( CAA E 28905514.0.0000.0005/2014).

### Autopsy procedures

**Fig. 1:**
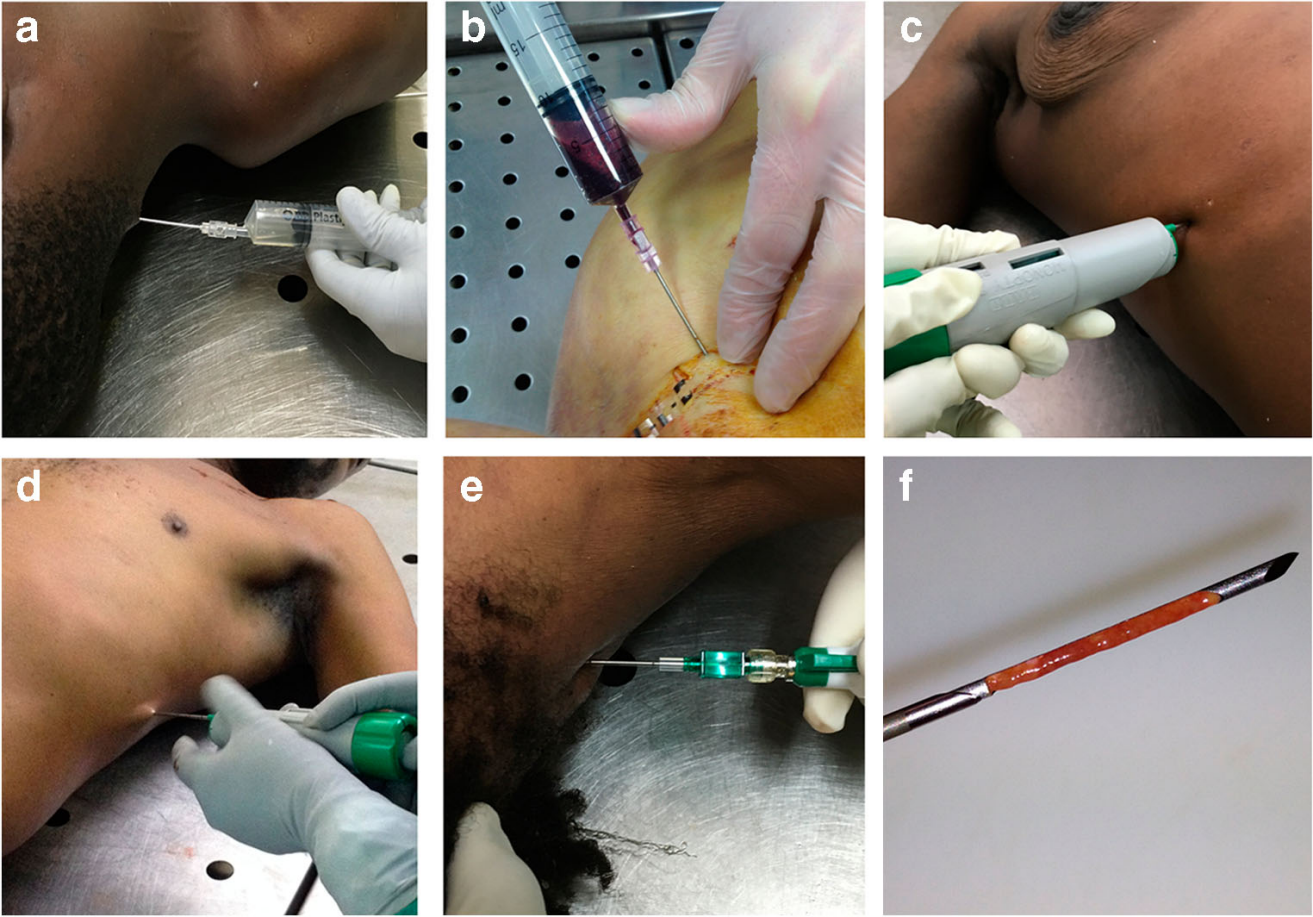
Representative figures of the sampling in a minimally invasive autopsy procedure. **a** Cerebrospinal fluid. **b** Peripheral blood. **c** Liver. **d** Lung. **e** Central nervous systembiopsy. **f** Example of a lung core obtained during minimally invasive autopsy procedure

The MIA procedure has been described in detail elsewhere [16]. The procedure included initial disinfection of the surface of the body followed by the collection of blood and cerebrospinal fluid (CSF), aiming to collect 20 mL of each fluid. It included the puncture of solid organs (liver, lungs and central nervous system [CNS]) using biopsy needles (14G–16G) and bone marrow (using a trephine needle) for microbiological and pathological analysis. In addition, the heart, spleen and kidneys were sampled for pathology examination. Representative figures of the sampling in a MIA procedure and an example of a lung core obtained during the procedure are shown in Fig. 1.

Immediately after the MIA, the CDA was conducted by a second pathologist not involved with the MIA and following a standardized protocol [17]. Histological and microbiological analyses were conducted in samples from the same viscera collected in the MIA and from any grossly identified lesions.

### Histological and microbiological analyses

A team of two pathologists (P.C., J.O.) and two microbiologists (J.C.H., M.J.M.) analysed the MIA samples blindly to any clinical information. All the samples were stained with haematoxylin and eosin and ancillary histochemical and/or immunohistochemical stains if required. Microbiological methods have been reported in detail else- where [18]. In brief, universal screening was performed for all cases, which included detection of *Plasmodium falciparum and Plasmodium* vivax by PCR, detection of antibodies against HIV-1/2 and bacterial/fungal cultures of CSF, blood, lungs, liver and CNS.

Additional microbiological screening applied to all HIV-positive cases included real-time PCR in CSF and CNS samples for Toxoplasma gondii, Mycobacterium tuberculosis, and Cryptococcus spp. and in lung samples for Pneumocystis jirovecii, Cryptococcus spp. and Mycobacterium tuberculosis. Other microorganisms were tested depending on the histological findings of the MIA or the CDA (e.g. Leishmania spp., Histoplasma capsulatum) [19, 20].

The pathology team in Manaus (A.P., M.F., L.F.) analysed the CDA samples following the same approach used for the analysis of the MIA samples, with the only exception that CDA tissues were not routinely cultured.

### Determination of the CoD

Once all the analyses of the MIA samples had been completed, a panel composed of a pathologist, a microbiologist and an internist evaluated all the data and assigned the MIA diagnosis. Details of the methodology for CoD determination have been described elsewhere [11]. Briefly, the MIA findings were used to assign the immediate condition leading to death. For the sake of the blind comparison MIA-CDA, no clinical information was used for the MIA diagnosis assignment, even if this could potentially result in a poorer concordance. A chain of conditions (up to four) was established following the most probable chronological sequence of events leading to death [21]. Fundamental diseases contributing to the death were classified as underlying conditions (e.g. HIV infection). In all cases, the immediate CoD and not the underlying disease was considered as the main CoD (e.g. disseminated histoplasmosis in an HIV-infected patient). Other conditions or concomitant infections unrelated to the chain of events were considered as other significant conditions.

The team from Manaus, which included three pathologists and an internist (A.P., M.F., L.F., M.L.), evaluated the data from the CDA and assigned the final CDA diagnosis using the same methodology. The CDA diagnosis integrated all the findings from the macroscopic, histological and microbiological analysis together with the clinical information and was considered the “gold standard” diagnosis.

All morbid conditions and any underlying diseases were codified following the International Classification of Diseases, tenth revision (ICD-10) [22], independently for the MIA and the CDA diagnoses. The CoD were classified into four major categories: (1) infectious diseases, (2) malignant tumours, (3) other diseases (including non-infectious cardiovascular or pulmonary diseases) and (4) non-conclusive.

### Review of the clinical charts

The available clinical information was reviewed and abstracted into a standardized questionnaire by one investigator from Manaus (A.P.). Up to five clinical diagnoses previously coded in ICD-10 registered in the medical record were extracted. In this list, we assumed that diagnosis number 1 was the principal diagnosis, and the remaining diagnoses were secondary. All CoD were divided into the four major categories of diseases. Diagnostic discrepancies were evaluated using the classification of Goldman et al. [23] modified by Battle et al. [24] and as nonclassifiable cases [25]. Major discrepancies were classified as class I or class II. Minor discrepancies were classified as classes III and IV, as previously described [8]. Correctly diagnosed patients were classified as class V. Class VI were non-classifiable cases.

### Statistical methods

The concordance between the MIA and the gold standard diagnosis (CDA) as well as the concordance between the clinical and the gold standard diagnosis were assessed by the Kappa statistic and interpreted as suggested by Landis and Koch [26]. The diagnostic accuracy of the MIA to identify the categories was evaluated as sensitivity, specificity, positive and negative predictive values and total percentage of cases correctly classified.

Additionally, the coincidence between the MIA and the CDA diagnoses, as well as the coincidence between the clinical and the CDA diagnoses for each case were assessed by comparing ICD-10 codes. The ICD-10 classifies diagnoses into nested classes of different hierarchical levels, where diseases or conditions are organized in chapters, blocks, and three character categories [21, 22]. Thus, a coincidence was classified as perfect when the ICD-10 codes were identical in chapter, block and three-character categories [21]. Coincidence was classified as moderate when the codes were within the same chapter and block, but there was a discrepancy in the three-character category and as low when the codes were within the same chapter, but there was a discrepancy in the block and three-character categories. When the MIA and the gold standard diagnoses were in a different chapter, the coincidence in diagnosis was classified as “none”.

Statistical analyses were performed using the Stata Software (Version 15, StataCorp 2017, College Station, TX, USA).

## Results

### General characteristics of the series

Coupled MIA and CDA were performed to 61 patients (39 males [64%] and 22 females [36%]). Fifty-nine of the patients were adults; two of them were maternal deaths (one woman who died during pregnancy and another who died during the puerperal period). The series included two children (a female and a male, both 13 years old). The median age of the overall group was 34.8 (range 13.9 to 81.5 years).

### Gold standard (CDA) diagnosis of CoD

A final CoD was identified in the CDA for all cases. The CDA classified 44/61 (72%) deaths as infectious (28 disseminated, eight pulmonary, seven CNS, and one liver infection). Eleven deaths (18%) were caused by malignancies (five liver cell carcinomas, six other malignancies), and 6/61 deaths (10%) were caused by other diseases. None of the maternal deaths was of a direct obstetric cause (one pulmonary thromboembolism complicating sickle cell anaemia and a liver cell carcinoma).

An underlying CoD was identified in the CDA in 51/61 of the cases (84%). Underlying diseases included HIV infection in 37 patients (61%). All of them tested positive for HIV antibodies (all being HIV-1). Hepatitis B virus (HBV) infection was identified in seven cases, three of them with an HDV(Hepatitis D virus)-HBV co-infection. In three patients, cirrhosis without aetiology was identified. One patient had sickle cell anaemia, one HTLV(Human T-lymphotropic virus) I-II infection and one systemic lupus erythematosus with glomerulonephritis.

### Concordance in disease categorization between the MIA and the CDA and accuracy of the MIA

A final cause of death was identified in the MIA in all cases. The MIA categorization of diseases agreed with the gold standard in 55/61 (90%) of the cases and showed a substantial concordance with the CDA categorization (Kappa = 0.780, 95% CI 0.608–0.946). Concordance in all categories was high, with a 100% (6/6) for other diseases, 93% (41/44) for infections and 73% (8/11) for the malignancies. From the discrepant cases, three out of 44 infections were classified as other diseases, and three out of eight malignancies were classified as infections.

Table 1 shows the sensitivity, specificity, the positive and negative predictive values for the major diagnostic categories, as well as the accuracy of the MIA diagnoses. Sensitivity and overall accuracy of the MIA were high for all categories, over 70% and 90%, respectively.

**Table 1:** Sensitivity, specificity, positive and negative predictive value (PPV and NPV) and accuracy of the MIA for the different diagnostic categories. Figures are percentages and 95% confidence intervals

Cause of death	Number	Sensitivity	Specificity	PPV	NPV	Correctly classified
Infectious diseases	44	93 (85,99)	82 (57,96)	93 (82,99)	82 (57,96)	90 (80,97)
Malignant tumours	11	73 (39,94)	100 (93,100)	100 (63,100)	94 (84,99)	95 (86,99)
Other diseases	6	100 (54,100)	95 (85,100)	67 (30,93)	100 (93,100)	95 (86,99)

Kappa (Std. Err.) 0.777—Substantial. 95% CI 0.608–0.946 *MIA* minimally invasive autopsy

### Coincidence between the MIA and the gold standard diagnoses

For the cases with no discrepancies (n = 55) between the MIA and the CDA diagnoses, we performed an additional analysis to evaluate the degree of coincidence (from perfect to none) between the specific ICD-10 diagnoses. Table 2 summarizes the coincidence in ICD-10 coding, in terms of identical chapter and block, according to disease category. The coincidence was perfect (i.e. the same specific disease, diagnosed as the CoD) in 47 of the 55 cases (85%). For the disease categories, this perfect level of coincidence was 67% for other diseases, being higher for infectious diseases and malignant tumours (over 85%, each). In the seven cases with moderate to low coincidence, the MIA failed to determine the organ or the microorganism (five cases of infectious diseases), the location of the carcinoma (one case) or the precise cardiovascular disease leading to death (one case of other diseases). In one case, the coincidence was considered none and included a pulmonary thromboembolism complicating a sickle cell anaemia crisis in which the MIA identified only a pulmonary infarct.

**Table 2:** Diagnostic coincidence within the 55 cases with concordant diagnosis between the MIA and the CDA

Disease group	Coincidence in diagnosis (MIA vs. CDA)					
	Perfect/almost perfect		Moderate or low		None	
	*n*	%(95% CI)	*N*	%(95%CI)	*n*	%(95% CI)
Overall (*n* = 55)	48	87 (66-88)	6	11 (4 -20)	1*	4 (0-8)
Infectious diseases (*n* =41)	37	90 (47-72)	4	10 (2-16)	0	0 (0-6)**
Malignant tumours (*n* = 8)	7	87 (5-22)	1	12 (0-9)	0	0 (0-6)**
Other diseases (*n* = 6)	4	67(2-16)	1	17 (0-9)	1	17 (0-9)

*MIA* minimally invasive autopsy, *CDA* complete diagnostic autopsy, *CI* confidence interval

* The case in which the coincidence was considered “none” is a sickle cell anaemia crisis that the MIA diagnosed as a pulmonary infarct.

** One-sided, 97.5% Confidence interval

Other significant diseases or co-infections were identified in 33/61 (54%) cases of the MIA and in 42/61 (69%) cases of the CDA, being coincident between the MIA and the CDA in 28 cases (46%).

Table S1 summarizes the CDA and MIA CoD, the underlying conditions and any other significant conditions identified at the CDA and the MIA for each individual patient, as well as the ICD-10 code for each diagnosis and coincidence between the MIA and the gold standard diagnosis.

### Aetiological agents and contribution of pathological and microbiological findings in infectious CoD

In 40/44 (91%) of the infection-related deaths, the aetiological agent was identified in the CDA. In the group of disseminated infections, seven patients had a milliary tuberculosis (6/7 HIV- positive), six had a disseminated histoplasmosis (all of them HIV-positive), five had a bacterial sepsis (three *Neisseria meningitidis, two Streptococcus pneumoniae* [one of the latter HIV-positive]), three had a disseminated cryptococcosis, three had a toxoplasmosis and two patients disseminated cytomegalovirus disease (all of them HIV-positive). Finally, one patient had disseminated leishmaniasis and one histological findings of haemorrhagic viral disease, although the agent was not identified after testing for dengue, yellow fever, leptospirosis and hantaviruses. The pulmonary infections included one tuberc ulosis, one pneumocystosis and two cases of cytomegaloviral pneumonia (all in HIV-positive patients); we also diagnosed two patients with pneumonia and one case with organizing pneumonia, but no agent was identified. The CNS infections included three toxoplasmosis, two cryptococcosis (all in HIV-positive patients) and two bacterial meningitis (one caused by N. *meningitidis* and one by Klebsiella *pneumoniae*).

The same microorganism identified in the gold standard CDA was identified in the MIA in 35 out of the 40 cases (87%). Table 3 shows the aetiological agents identified in the CDA and in the MIA.

**Table 3:** List of aetiological agents identified in the complete diagnostic autopsy, the minimally invasive autopsy and in both methods, expressed in number of cases

Aetiological agents	Complete diagnostic autopsy (CDA, gold standard)	Minimally invasive autopsy (MIA)	Microorganism identified by both methods (MIA and CDA)
Disseminated infections	**27**	**27**	**27**
*Mycobacterium tuberculosis*	7	7	7
*Histoplama capsulatum*	6	6	6
*Cryptococcus spp.*	3	3	3
*Toxoplasma gondii*	3	3	3
*Neisseria meningitidis*	3	3	3
*Cytomegalovirus*	2	2	2
*Streptococcus pneumoniae*	2	2	2
*Leishmania spp.*	1	1	1
Pulmonary infections	**5**	**5**	**5**
*Cytomegalovirus*	2	2	2
*Pneumocystis jirovecii*	1	1	1
*Pseudomonas aeruginosa*	1	1	1
*Mycobacterium tuberculosis*	1	1	1
Central nervous system infections	**7**	**2**	**2**
*Toxoplasma gondii*	3	1	1
*Cryptococcus spp.*	2	0	0
*Neisseria meningitidis*	1	1	1
*Klebsiella pneumoniae*	1	0	0
Other infections	1	1	1
Viral hepatitis A	1	1	1
Total	**40**	**35**	**35**

The entries that were marked in bold are the main groups of different diagnosis *No agent was identified in three pulmonary infections and one disseminated infection

Among the 44 infection-related deaths, the pathological findings alone were sufficient for the cause of death diagnosis in 12 cases (27%) of the MIA and in 5 cases (11%) of the CDA. In 23 cases (52%) of the MIA and in 39 (89%) cases of the CDA, the pathological and the microbiological findings contributed equally, with microbiology providing information on the aetiological agent. Finally, in 9 cases of the MIA (21%), the pathological findings were non-conclusive and the diagnosis was based on the microbiological analysis.

### Clinico-pathological discrepancies

The clinical categorization of disease showed a fair concordance with the CDA categorization (Kappa = 0.311, 95% CI 0.071–0.552). The sensitivity of the clinical diagnosis was 93% for infectious diseases (95% CI 81–93%), 67% for other diseases (95% CI 22–96%) and 0% for malignant tumours (95% CI 0–28%).

Overall, a diagnostic discrepancy was determined in 31/61 (51%) of the cases. A major diagnostic discrepancy was detected in 30 (49%) of deaths: 25 (41%) were classified as class I, and five (8%) as class II. A minor diagnostic discrepancy (class III) was identified in one case. In 30 cases (49%), there was complete agreement between the clinical and the autopsy diagnoses (class V). Twenty-one of the 30 major discordances (70%), occurred in infections, six (20%) in malignancies and three (10%) in other diseases. No statistical differences in terms of percentage of major errors were observed between the main diagnostic categories (*p* = 0.142). No association was observed between demographic data, clinical symptoms at admission and biochemical parameters and type of clinicopathological discrepancies.

## Discussion

To our knowledge, this is the first study reporting the validity of a standardized MIA for CoD determination and including the evaluation of the clinico-pathological discrepancies in the Brazilian Amazon. The validation study shows a substantial degree of concordance (90%; kappa value of 0.78) between the MIA and the gold standard diagnoses. The concordance was remarkably high in all disease categories, particularly for infectious diseases. These results are consistent with the reported concordance in a series of adults in Mozambique [11] and show that the MIA can be an adequate alternative in LMIC settings, where mortality statistics are poor.

Infectious diseases, representing over 70% of all deaths, and HIV infection (61% of all cases) were as prevalent as in our previous study in Mozambique [11]. These infections include endemic diseases such as histoplasmosis and leishmaniasis, as well as microorganisms such as N. *meningitidis* that were not identified in previous validation studies [11, 13, 14]. Importantly, in 87% of the cases, the aetiological microorganism was identified in the MIA. Interestingly, all five missed aetiological agents, not identified at MIA and detected with molecular methods in the CDA-obtained tissues, were CNS infections. In three cases, this was due to the absence of meningeal membranes in the MIA sample (two meningitis), and in two cases, the histological lesion and the microorganism were absent in MIA sample (two toxoplasmosis). These findings also indicate that the CSF collected, during MIA, cannot be sufficient on its own to identify infectious agent affecting the CNS. Future MIA protocols may benefit from an increase in the number of CNS samples in order to increase the yield of the microbiological and histological results in meningeal infections.

Similar to other settings, M. *tuberculosis* was the most frequent fatal opportunistic infection among HIV-positive patients in Manaus. *Cryptococcus* spp. was also a frequent cause of death [11, 27]. In keeping with other studies in Latin America [28], histoplasmosis, an endemic fungal infection, was the second most frequent infectious disease identified as CoD in the HIV-positive patients from our series. In a previous autopsy study conducted in the same setting and including HIV patients from 1996 to 2003, tuberculosis (28%) and histoplasmosis (17%) were also the two most frequent causes of death [29]. These results are similar to other studies in Latin America, including Brazil, reporting high incidence and mortality of histoplasmosis especially in HIV-positive patients [30–32]. The high frequency of histoplasmosis in this area indicates that this infection needs to be considered as part of the differential diagnosis early during clinical management of HIV-positive patients.

Bacterial pneumonia was an infrequent CoD in the present series in HIV-positive patients, which contrasts with previous reports [29, 33]. These results may reflect an improvement in the management of bacterial infections in HIV-positive patients [29]. Finally, blood samples obtained during MIA, allowed us to identify all HBV infections (seven cases), which in three cases had an associated HDV co-infection. In addition, an HTLV I-II co-infection was also identified.

Hepatocellular carcinoma was the most frequent malignancy in this series; all were diagnosed in HIV negative patients. Four out of the five hepatocellular carcinomas identified were associated with HBV infection. Thus, although HCV infection is now the most frequent risk factor associated with HCC in South America, including Brazil [34, 35], HBV infection remains an important risk factor for HCC in some regions, including the study area. The high accuracy of MIA to detect malignant tumours is consistent with previous data [11]. However, in three patients, a malignant tumour identified in the CDA was not diagnosed in the MIA. All these tumours missed at MIA were localized tumours, being out of the target regions of the MIA (hepatocarcinoma, one large B cell lymphoma and one gastric adenocarcinoma).

Clinico-pathological discrepancies were identified in 51% of the cases (31/61), with most of the discrepancies being major errors (30 cases; 49% of the overall series). Importantly, in most patients (41%), a change in the clinical management could have significantly modified the prognosis (type I errors). The percentage of discrepancies observed in this series is similar to the data of tertiary hospital of Mozambique and is higher than in most studies based on unselected hospital patients [18, 23–25].

The main limitation of our study is that it was conducted in a referral tertiary care centre focused on infectious diseases, in a cohort of cases with a high HIV prevalence, not representative of the population. This could limit the extrapolation of the findings to other health facilities and to high-income countries, where HIV and infections may be less prevalent and cardiovascular diseases and malignant neoplasms are frequent causes of death. However, the present study was conducted in a completely different geographic area compared to our previous studies, conducted in sub-Saharan Africa [11, 12, 14], indicating that MIA may provide robust mortality data irrespectively of the region.

In conclusion, in this series of adult deaths from the Brazilian Amazon, the MIA showed a substantial performance for CoD determination. The results confirm that the MIA is a valid tool, comparable to the gold standard methodology, particularly for infectious diseases, and that it can be reliably considered an adequate proxy for the CDA for CoD surveillance and quality improvement of clinical practice. The high accuracy of the MIA to detect infectious diseases indicates that the procedure has a potential use to detect infectious diseases outbreaks and emerging diseases in sentinel sites. Major c linicopathological discrepancies remain significantly high (41%), even in a referral hospital with adequate diagnostic resources and specialized physicians, as to justify the need for post-mortem procedures, which may help to improve clinical practice in LMIC.

## Notes

**Publisher’s note** Springer Nature remains neutral with regard to jurisdictional claims in published maps and institutional affiliations.

## References

[1] Wang H, Abajobir AA, Abate KH, Abbafati C, Abbas KM, Abd-Allah F, Abera SF, Abraha HN, Abu-Raddad LJ, Abu-Rmeileh NME, Adedeji IA, Adedoyin RA, Adetifa IMO, Adetokunboh O, Afshin A, Aggarwal R, Agrawal A, Agrawal S, Ahmad Kiadaliri A, Ahmed MB, Aichour MTE, Aichour AN, Aichour I, Aiyar S, Akanda AS, Akinyemiju TF, Akseer N, al Lami FH, Alabed S, Alahdab F, al-Aly Z, Alam K, Alam N, Alasfoor D, Aldridge RW, Alene KA, al-Eyadhy A, Alhabib S, Ali R, Alizadeh-Navaei R, Aljunid SM, Alkaabi JM, Alkerwi A’, Alla F, Allam SD, Allebeck P, al-Raddadi R, Alsharif U, Altirkawi KA, Alvis-Guzman N, Amare AT, Ameh EA, Amini E, Ammar W, Amoako YA, Anber N, Andrei CL, Androudi S, Ansari H, Ansha MG, Antonio CAT, Anwari P, Ärnlöv J, Arora M, Artaman A, Aryal KK, Asayesh H, Asgedom SW, Asghar RJ, Assadi R, Assaye AM, Atey TM, Atre SR, Avila-Burgos L, Avokpaho EFGA, Awasthi A, Babalola TK, Bacha U, Badawi A, Balakrishnan K, Balalla S, Barac A, Barber RM, Barboza MA, Barker-Collo SL, Bärnighausen T, Barquera S, Barregard L, Barrero LH, Baune BT, Bazargan-Hejazi S, Bedi N, Beghi E, Béjot Y, Bekele BB, Bell ML, Bello AK, Bennett DA, Bennett JR, Bensenor IM, Benson J, Berhane A, Berhe DF, Bernabé E, Beuran M, Beyene AS, Bhala N, Bhansali A, Bhaumik S, Bhutta ZA, Bicer BK, Bidgoli HH, Bikbov B, Birungi C, Biryukov S, Bisanzio D, Bizuayehu HM, Bjerregaard P, Blosser CD, Boneya DJ, Boufous S, Bourne RRA, Brazinova A, Breitborde NJK, Brenner H, Brugha TS, Bukhman G, Bulto LNB, Bumgarner BR, Burch M, Butt ZA, Cahill LE, Cahuana-Hurtado L, Campos-Nonato IR, Car J, Car M, Cárdenas R, Carpenter DO, Carrero JJ, Carter A, Castañeda-Orjuela CA, Castro FF, Castro RE, Catalá-López F, Chen H, Chiang PPC, Chibalabala M, Chisumpa VH, Chitheer AA, Choi JYJ, Christensen H, Christopher DJ, Ciobanu LG, Cirillo M, Cohen AJ, Colquhoun SM, Coresh J, Criqui MH, Cromwell EA, Crump JA, Dandona L, Dandona R, Dargan PI, das Neves J, Davey G, Davitoiu DV, Davletov K, de Courten B, de Leo D, Degenhardt L, Deiparine S, Dellavalle RP, Deribe K, Deribew A, Des Jarlais DC, Dey S, Dharmaratne SD, Dherani MK, Diaz-Torné C, Ding EL, Dixit P, Djalalinia S, Do HP, Doku DT, Donnelly CA, dos Santos KPB, Douwes-Schultz D, Driscoll TR, Duan L, Dubey M, Duncan BB, Dwivedi LK, Ebrahimi H, el Bcheraoui C, Ellingsen CL, Enayati A, Endries AY, Ermakov SP, Eshetie S, Eshrati B, Eskandarieh S, Esteghamati A, Estep K, Fanuel FBB, Faro A, Farvid MS, Farzadfar F, Feigin VL, Fereshtehnejad SM, Fernandes JG, Fernandes JC, Feyissa TR, Filip I, Fischer F, Foigt N, Foreman KJ, Frank T, Franklin RC, Fraser M, Friedman J, Frostad JJ, Fullman N, Fürst T, Furtado JM, Futran ND, Gakidou E, Gambashidze K, Gamkrelidze A, Gankpé FG, Garcia-Basteiro AL, Gebregergs GB, Gebrehiwot TT, Gebrekidan KG, Gebremichael MW, Gelaye AA, Geleijnse JM, Gemechu BL, Gemechu KS, Genova-Maleras R, Gesesew HA, Gething PW, Gibney KB, Gill PS, Gillum RF, Giref AZ, Girma BW, Giussani G, Goenka S, Gomez B, Gona PN, Gopalani SV, Goulart AC, Graetz N, Gugnani HC, Gupta PC, Gupta R, Gupta R, Gupta T, Gupta V, Haagsma JA, Hafezi-Nejad N, Hakuzimana A, Halasa YA, Hamadeh RR, Hambisa MT, Hamidi S, Hammami M, Hancock J, Handal AJ, Hankey GJ, Hao Y, Harb HL, Hareri HA, Harikrishnan S, Haro JM, Hassanvand MS, Havmoeller R, Hay RJ, Hay SI, He F, Heredia-Pi IB, Herteliu C, Hilawe EH, Hoek HW, Horita N, Hosgood HD, Hostiuc S, Hotez PJ, Hoy DG, Hsairi M, Htet AS, Hu G, Huang JJ, Huang H, Iburg KM, Igumbor EU, Ileanu BV, Inoue M, Irenso AA, Irvine CMS, Islam SMS, Islam N, Jacobsen KH, Jaenisch T, Jahanmehr N, Jakovljevic MB, Javanbakht M, Jayatilleke AU, Jeemon P, Jensen PN, Jha V, Jin Y, John D, John O, Johnson SC, Jonas JB, Jürisson M, Kabir Z, Kadel R, Kahsay A, Kalkonde Y, Kamal R, Kan H, Karch A, Karema CK, Karimi SM, Karthikeyan G, Kasaeian A, Kassaw NA, Kassebaum NJ, Kastor A, Katikireddi SV, Kaul A, Kawakami N, Kazanjan K, Keiyoro PN, Kelbore SG, Kemp AH, Kengne AP, Keren A, Kereselidze M, Kesavachandran CN, Ketema EB, Khader YS, Khalil IA, Khan EA, Khan G, Khang YH, Khera S, Khoja ATA, Khosravi MH, Kibret GD, Kieling C, Kim YJ, Kim CI, Kim D, Kim P, Kim S, Kimokoti RW, Kinfu Y, Kishawi S, Kissoon N, Kivimaki M, Knudsen AK, Kokubo Y, Kopec JA, Kosen S, Koul PA, Koyanagi A, Kravchenko M, Krohn KJ, Kuate Defo B, Kuipers EJ, Kulikoff XR, Kulkarni VS, Kumar GA, Kumar P, Kumsa FA, Kutz M, Lachat C, Lagat AK, Lager ACJ, Lal DK, Lalloo R, Lambert N, Lan Q, Lansingh VC, Larson HJ, Larsson A, Laryea DO, Lavados PM, Laxmaiah A, Lee PH, Leigh J, Leung J, Leung R, Levi M, Li Y, Liao Y, Liben ML, Lim SS, Linn S, Lipshultz SE, Liu S, Lodha R, Logroscino G, Lorch SA, Lorkowski S, Lotufo PA, Lozano R, Lunevicius R, Lyons RA, Ma S, Macarayan ERK, Machado IE, Mackay MT, Magdy Abd el Razek M, Magis-Rodriguez C, Mahdavi M, Majdan M, Majdzadeh R, Majeed A, Malekzadeh R, Malhotra R, Malta DC, Mantovani LG, Manyazewal T, Mapoma CC, Marczak LB, Marks GB, Martin EA, Martinez-Raga J, Martins-Melo FR, Massano J, Maulik PK, Mayosi BM, Mazidi M, McAlinden C, McGarvey ST, McGrath JJ, McKee M, Mehata S, Mehndiratta MM, Mehta KM, Meier T, Mekonnen TC, Meles KG, Memiah P, Memish ZA, Mendoza W, Mengesha MM, Mengistie MA, Mengistu DT, Menon GR, Menota BG, Mensah GA, Meretoja TJ, Meretoja A, Mezgebe HB, Micha R, Mikesell J, Miller TR, Mills EJ, Minnig S, Mirarefin M, Mirrakhimov EM, Misganaw A, Mishra SR, Mohammad KA, Mohammadi A, Mohammed KE, Mohammed S, Mohan MBV, Mohanty SK, Mokdad AH, Mollenkopf SK, Molokhia M, Monasta L, Montañez Hernandez JC, Montico M, Mooney MD, Moore AR, Moradi-Lakeh M, Moraga P, Morawska L, Mori R, Morrison SD, Mruts KB, Mueller UO, Mullany E, Muller K, Murthy GVS, Murthy S, Musa KI, Nachega JB, Nagata C, Nagel G, Naghavi M, Naidoo KS, Nanda L, Nangia V, Nascimento BR, Natarajan G, Negoi I, Nguyen CT, Nguyen QL, Nguyen TH, Nguyen G, Ningrum DNA, Nisar MI, Nomura M, Nong VM, Norheim OF, Norrving B, Noubiap JJN, Nyakarahuka L, O’Donnell MJ, Obermeyer CM, Ogbo FA, Oh IH, Okoro A, Oladimeji O, Olagunju AT, Olusanya BO, Olusanya JO, Oren E, Ortiz A, Osgood-Zimmerman A, Ota E, Owolabi MO, Oyekale AS, PA M, Pacella RE, Pakhale S, Pana A, Panda BK, Panda-Jonas S, Park EK, Parsaeian M, Patel T, Patten SB, Patton GC, Paudel D, Pereira DM, Perez-Padilla R, Perez-Ruiz F, Perico N, Pervaiz A, Pesudovs K, Peterson CB, Petri WA, Petzold M, Phillips MR, Piel FB, Pigott DM, Pishgar F, Plass D, Polinder S, Popova S, Postma MJ, Poulton RG, Pourmalek F, Prasad N, Purwar M, Qorbani M, Quintanilla BPA, Rabiee RHS, Radfar A, Rafay A, Rahimi-Movaghar A, Rahimi-Movaghar V, Rahman MHU, Rahman SU, Rahman M, Rai RK, Rajsic S, Ram U, Rana SM, Ranabhat CL, Rao PV, Rawaf S, Ray SE, Rego MAS, Rehm J, Reiner RC, Remuzzi G, Renzaho AMN, Resnikoff S, Rezaei S, Rezai MS, Ribeiro AL, Rivas JC, Rokni MB, Ronfani L, Roshandel G, Roth GA, Rothenbacher D, Roy A, Rubagotti E, Ruhago GM, Saadat S, Sabde YD, Sachdev PS, Sadat N, Safdarian M, Safi S, Safiri S, Sagar R, Sahathevan R, Sahebkar A, Sahraian MA, Salama J, Salamati P, Salomon JA, Salvi SS, Samy AM, Sanabria JR, Sanchez-Niño MD, Santos IS, Santric Milicevic MM, Sarmiento-Suarez R, Sartorius B, Satpathy M, Sawhney M, Saxena S, Saylan MI, Schmidt MI, Schneider IJC, Schulhofer-Wohl S, Schutte AE, Schwebel DC, Schwendicke F, Seedat S, Seid AM, Sepanlou SG, Servan-Mori EE, Shackelford KA, Shaheen A, Shahraz S, Shaikh MA, Shamsipour M, Shamsizadeh M, Sharma J, Sharma R, She J, Shen J, Shetty BP, Shi P, Shibuya K, Shifa GT, Shigematsu M, Shiri R, Shiue I, Shrime MG, Sigfusdottir ID, Silberberg DH, Silpakit N, Silva DAS, Silva JP, Silveira DGA, Sindi S, Singh JA, Singh PK, Singh A, Singh V, Sinha DN, Skarbek KAK, Skiadaresi E, Sligar A, Smith DL, Sobaih BHA, Sobngwi E, Soneji S, Soriano JB, Sreeramareddy CT, Srinivasan V, Stathopoulou V, Steel N, Stein DJ, Steiner C, Stöckl H, Stokes MA, Strong M, Sufiyan MB, Suliankatchi RA, Sunguya BF, Sur PJ, Swaminathan S, Sykes BL, Szoeke CEI, Tabarés-Seisdedos R, Tadakamadla SK, Tadese F, Tandon N, Tanne D, Tarajia M, Tavakkoli M, Taveira N, Tehrani-Banihashemi A, Tekelab T, Tekle DY, Temsah MH, Terkawi AS, Tesema CL, Tesssema B, Theis A, Thomas N, Thompson AH, Thomson AJ, Thrift AG, Tiruye TY, Tobe-Gai R, Tonelli M, Topor-Madry R, Topouzis F, Tortajada M, Tran BX, Truelsen T, Trujillo U, Tsilimparis N, Tuem KB, Tuzcu EM, Tyrovolas S, Ukwaja KN, Undurraga EA, Uthman OA, Uzochukwu BSC, van Boven JFM, Varakin YY, Varughese S, Va sank ari T , Vasconcelos AMN, Ve lasquez I M, Venketasubramanian N, Vidavalur R, Violante FS, Vishnu A, Vladimirov SK, Vlassov VV, Vollset SE, Vos T, Waid JL, Wakayo T, Wang YP, Weichenthal S, Weiderpass E, Weintraub RG, Werdecker A, Wesana J, Wijeratne T, Wilkinson JD, Wiysonge CS, Woldeyes BG, Wolfe CDA, Workicho A, Workie SB, Xavier D, Xu G, Yaghoubi M, Yakob B, Yalew AZ, Yan LL, Yano Y, Yaseri M, Ye P, Yimam HH, Yip P, Yirsaw BD, Yonemoto N, Yoon SJ, Yotebieng M, Younis MZ, Zaidi Z, Zaki MES, Zeeb H, Zenebe ZM, Zerfu TA, Zhang AL, Zhang X, Zodpey S, Zuhlke LJ, Lopez AD, Murray CJL (2017) Global, regional, and national under-5 mortality, adult mortality, age-specific mortality, and life expectancy, 1970–2016: a systematic analysis for the Global Burden of Disease Study 2016. Lancet 390:1084–1150 28919115 10.1016/S0140-6736(17)31833-0 PMC5605514

[2] Blokker BM, Weustink AC, Hunink MGM, Oosterhuis JW (2017) Autopsy rates in the Netherlands: 35 years of decline. PLoS One 12:e0178200 28617835 10.1371/journal.pone.0178200 PMC5472266

[3] Shojania KG, Burton EC, McDonald KM et al (2003) Changes in rates of autopsy-detected diagnostic errors over time: a systematic review. JAMA 289:2849–2856 12783916 10.1001/jama.289.21.2849

[4] Burton JL, Underwood J (2007) Clinical, educational, and epidemiological value of autopsy. Lancet 369:1471–1480 17467518 10.1016/S0140-6736(07)60376-6

[5] Turnbull A, Osborn M, Nicholas N (2015) Hospital autopsy: endangered or extinct? J Clin Pathol 68:601–604 26076965 10.1136/jclinpath-2014-202700 PMC4518760

[6] Loughrey MB, McCluggage WG, Toner PG (2000) The declining autopsy rate and clinicians’ attitudes. Ulster Med J 69:83–89 11196736 PMC2449188

[7] Oluwasola OA, Fawole OI, Otegbayo AJ, Ogun GO, Adebamowo CA, Bamigboye AE (2009) The autopsy knowledge, attitude, and perceptions of doctors and relatives of the deceased. Arch Pathol Lab Med 133:78–82 19123741 10.5858/133.1.78

[8] Ordi J, Ismail MR, Carrilho C et al (2009) Clinico-pathological discrepancies in the diagnosis of causes of maternal death in sub-Saharan Africa: retrospective analysis. PLoSMed 6:e1000036 10.1371/journal.pmed.1000036 PMC2646780 19243215

[9] Kuijpers CC, Fronczek J, Van De Goot FR et al (2014) The value of autopsies in the era of high-tech medicine: discrepant findings persist. J Clin Pathol 67:512–519 24596140 10.1136/jclinpath-2013-202122

[10] Sonnemans LJP, Kubat B, Prokop M, Klein WM (2018) Can virtual autopsy with postmortem CT improve clinical diagnosis of cause of death? A retrospective observational cohort study in a Dutch tertiary referral centre. BMJ Open 8:e018834 10.1136/bmjopen-2017-018834 PMC5857682 29549202

[cit0011] 11. Castillo P, Martinez MJ, Ussene E et al (2016) Validity of a minimally invasive autopsy for cause of death determination in adults in Mozambique: an observational study. PLoS Med 13:e1002171 27875530 10.1371/journal.pmed.1002171 PMC5119723

[12] Menendez C, Castillo P, Martínez MJ, Jordao D, Lovane L, Ismail MR, Carrilho C, Lorenzoni C, Fernandes F, Nhampossa T, Hurtado JC, Navarro M, Casas I, Santos Ritchie P, Bandeira S, Mocumbi S, Jaze Z, Mabota F, Munguambe K, Maixenchs M, Sanz A, Mandomando I, Nadal A, Goncé A, Muñoz-Almagro C, Quintó L, Vila J, Macete E, Alonso P, Ordi J, Bassat Q (2017) Validity of a minimally invasive autopsy for cause of death determination in stillborn babies and neonates in Mozambique: an observational study. PLoS Med 14:e1002318 28632735 10.1371/journal.pmed.1002318 PMC5478138

[13] Castillo P, Hurtado JC, Martínez MJ, Jordao D, Lovane L, Ismail MR, Carrilho C, Lorenzoni C, Fernandes F, Mocumbi S, Jaze ZO, Mabota F, Cossa A, Mandomando I, Cisteró P, Mayor A, Navarro M, Casas I, Vila J, Maixenchs M, Munguambe K, Sanz A, Quintó L, Macete E, Alonso P, Bassat Q, Ordi J, Menéndez C (2017) Validity of a minimally invasive autopsy for cause of death determination in maternal deaths in Mozambique: an observational study. PLoS Med 14:e1002431 29117196 10.1371/journal.pmed.1002431 PMC5695595

[14] Bassat Q, Castillo P, Martínez MJ, Jordao D, Lovane L, Hurtado JC, Nhampossa T, Santos Ritchie P, Bandeira S, Sambo C, Chicamba V, Ismail MR, Carrilho C, Lorenzoni C, Fernandes F, Cisteró P, Mayor A, Cossa A, Mandomando I, Navarro M, Casas I, Vila J, Munguambe K, Maixenchs M, Sanz A, Quintó L, Macete E, Alonso P, Menéndez C, Ordi J (2017) Validity of a minimally invasive autopsy tool for cause of death determination in pediatric deaths in Mozambique: an observational study. PLoS Med 14: e1002317 28632739 10.1371/journal.pmed.1002317 PMC5478091

[15] Instituto Brasileiro de Geografia e Estadística Brazilian demographic census. Brazil; 2016 https://cidades.ibge.gov.br/xtras/ perfil.php?lang=&codmun=130260&s earch= amazonas% 7Cmanaus (accessed 23 September 2017)

[16] Castillo P, Ussene E, Ismail MR, Jordao D, Lovane L, Carrilho C, Lorenzoni C, Lacerda MV, Palhares A, Rodríguez-Carunchio L, Martínez MJ, Vila J, Bassat Q, Menéndez C, Ordi J (2015) Pathological methods applied to the investigation of causes of death in developing countries: minimally invasive autopsy approach. PLoSOne 10:e0132057 10.1371/journal.pone.0132057 PMC4488344 26126191

[17] Hutchins GM (1994) Practice guidelines for autopsy pathology. Autopsy performance. Autopsy Committee of the College of American Pathologists. Arch Pathol Lab Med 118:19–25 8285830

[18] Martinez MJ, Massora S, Mandomando I et al (2016) Infectious cause of death determination using minimally invasive autopsies in developing countries. Diagn Microbiol Infect Dis 84:80–86 26508103 10.1016/j.diagmicrobio.2015.10.002

[19] Da Silva RM, Da Silva Neto JR, Santos CS et al (2015) Fluorescent in situ hybridization of pre-incubated blood culture material for the rapid diagnosis of histoplasmosis. Med Mycol 53:160–164 25537280 10.1093/mmy/myu080

[20] Koepsell SA, Hinrichs SH, Iwen PC (2012) Applying a real-time PCR assay for Histoplasma capsulatum to clinically relevant formalin-fixed paraffin-embedded human tissue. J Clin Microbiol 50:3395–3397 22855519 10.1128/JCM.01705-12 PMC3457455

[21] World Health Organization International classification of diseases (ICD-10) volume 2 instruction manual. 5th ed. Geneva: World Health Organization, https://es.scribd.com/document/318850076/ ICD-10-Volume-2-en-2016 (accessed 11 March 2019)

[22] World Health Organization International classification of diseases (ICD-10): international statistica classification of diseases and related health problems. 10th revision. 5th ed. Geneva: World Health Organization, http://apps.who.int/classifications/icd10/browse/2016/en (2016, accessed 15 May 2018)

[23] Goldman L, Sayson R, Robbins S, Cohn LH, Bettmann M, Weisberg M (1983) The value of the autopsy in three medical eras. NEnglJMed 308:1000–1005 10.1056/NEJM198304283081704 6835306

[24] Battle RM, Pathak D, Humble CG, Key CR, Vanatta PR, Hill RB, Anderson RE (1987) Factors influencing discrepancies between premortem and postmortem diagnoses. JAMA 258:339–344 3599326

[25] Grinberg LT, Ferraz da Silva LF, Galtarossa Xavier AC, Nascimento Saldiva PH, Mauad T (2008) Clinico-pathological discrepancies in the diagnoses of solid malignancies. Pathol Res Pract 204:867–873 18755553 10.1016/j.prp.2008.07.001

[26] Landis JR, Koch GG (1977) The measurement of observer agreement for categorical data. Biometrics 33:159–174 843571

[27] Békondi C, Bernede C, Passone N, Minssart P, Kamalo C, Mbolidi D, Germani Y (2006) Primary and opportunistic pathogens associated with meningitis in adults in Bangui, Central African Republic, in relation to human immunodeficiency virus serostatus. Int J Infect Dis 10:387–395 16473538 10.1016/j.ijid.2005.07.004

[28] Adenis A, Nacher M, Hanf M, Vantilcke V, Boukhari R, Blachet D, Demar M, Aznar C, Carme B, Couppie P (2014) HIV-associated histoplasmosis early mortality and incidence trends: from neglect to priority. PLoS Negl Trop Dis 8:e3100 25144374 10.1371/journal.pntd.0003100 PMC4140672

[29] de Souza SLS, Feitoza PVS, de Araújo JR et al Causes of death among patients with acquired immunodeficiency syndrome autopsied at the Tropical Medicine Foundation of Amazonas. Rev Soc Bras Med Trop 41:247–251 18719803 10.1590/s0037-86822008000300005

[30] da Silva Ferreira B, de Araújo Filho JA, Matos Pereira N, de Miranda Godoy L, Borges Lamounier B, Dias Nunes E, Espíndola Rosa L (2017) Disseminated histoplasmosis in AIDS patients: an urban disease. Experience in a metropolis in the middle east of Brasil. Infez Med 25:258–262 28956543

[31] Adenis AA, Valdes A, Cropet C, McCotter OZ, Derado G, Couppie P, Chiller T, Nacher M (2018) Burden of HIV-associated histoplasmosis compared with tuberculosis in Latin America: a modelling study. Lancet Infect Dis 18:1150–1159 30146320 10.1016/S1473-3099(18)30354-2 PMC6746313

[32] Pasqualotto AC, Quieroz-Telles F (2018) Histoplasmosis dethrones tuberculosis in Latin America. Lancet Infect Dis 18:1058–1060 30146319 10.1016/S1473-3099(18)30373-6

[33] Lanjewar DN (2011) The spectrum of clinical and pathological manifestations of AIDS in a consecutive series of 236 autopsied cases in Mumbai, India. Pathol Res Int 2011:547618 10.4061/2011/547618 PMC3108477 21660276

[34] de Martel C, Maucort-Boulch D, Plummer M, Franceschi S (2015) World-wide relative contribution of hepatitis B and C viruses in hepatocellular carcinoma. Hepatology 62:1190–1200 26146815 10.1002/hep.27969 PMC5019261

[35] Debes JD, Chan AJ, Balderramo D, Kikuchi L, Gonzalez Ballerga E, Prieto JE, Tapias M, Idrovo V, Davalos MB, Cairo F, Barreyro FJ, Paredes S, Hernandez N, Avendaño K, Diaz Ferrer J, Yang JD, Carrera E, Garcia JA, Mattos AZ, Hirsch BS, Gonçalves PT, Carrilho FJ, Roberts LR (2018) Hepatocellular carcinoma in South America: evaluation of risk factors, demographics and therapy. Liver Int 38:136–143 28640517 10.1111/liv.13502

