# Supplementary material for: Performance of the minimally invasive autopsy tool for cause of death determination in adult deaths from the Brazilian Amazon: an observational study
Source: Virchows Arch. 2019 Jun 14;475(5):649–58. doi: 10.1007/s00428-019-02602-z (PMC6861203; doi:10.1007/s00428-019-02602-z)
Supplement: Supplementary file 1 [file VA-2019-s00428-019-02602-z-s1.docx]

|  |  | **Gold standard diagnosis (complete diagnostic autopsy [CDA])** | | | | | | | **Minimally invasive autopsy (MIA) diagnosis** | | | | | | |
| --- | --- | --- | --- | --- | --- | --- | --- | --- | --- | --- | --- | --- | --- | --- | --- |
| **Case** | **Age** | **Sex** | **CDA Diagnosis** | **ICD-10** | **Underlying condition** | **ICD-10** | **Other significant conditions or coinfections** | **ICD-10** | **MIA diagnosis** | **ICD-10** | **Underlying condition** | **ICD-10** | **Other significant conditions or coinfections** | **ICD-10** | **Coincidence**  **ICD-10** |
| **INFECTIOUS DISEASES** | | | |  |  |  |  |  |  |  |  |  |  |  |  |
| **Disseminated infections** | | | | | |  |  |  |  |  |  |  |  |  |  |
| ***Mycobacterium tuberculosis*** | | | | | |  |  |  |  |  |  |  |  |  |  |
| 1 | 31 | F | Milliary tuberculosis | A19 | HIV | B20 |  |  | Pulmonary tuberculosis | A15 | HIV | B20 |  |  | Moderate |
| 6 | 29 | M | Milliary tuberculosis | A19 | HIV | B20 | *T. gondii* | B58 | Pulmonary tuberculosis | A15 | HIV | B20 | *T. gondii* | B58 | Moderate |
| 16 | 52 | M | Milliary tuberculosis | A19 | HIV | B20 | *T. gondii* | B58 | Milliary tuberculosis | A19 | HIV | B20 | *T. gondii* | B58 | Perfect |
| 24 | 25 | F | Milliary tuberculosis | A19 | HIV | B20 |  |  | Milliary tuberculosis | A19 | HIV | B20 |  |  | Perfect |
| 35 | 39 | F | Milliary tuberculosis | A19 | HIV | B20 |  |  | Milliary tuberculosis | A19 | HIV | B20 |  |  | Perfect |
| 58 | 31 | M | Milliary tuberculosis | A19 | HIV | B20 | CMV | B25.0 | Milliary tuberculosis | A19 | HIV | B20 | CMV | B25.0 | Perfect |
| 3 | 76 | M | Milliary tuberculosis | A19 |  | B20 |  |  | Milliary tuberculosis | A19 |  | B20 |  |  | Perfect |
| ***Histoplasma capsulatum*** | | | |  |  |  |  |  |  |  |  |  |  |  |  |
| 23 | 48 | M | Disseminated infection (*H. capsulatum*) | B39.3 | HIV | B20 | *E. coli*, CMV, *T. gondii* | B58.9 | Disseminated infection (*H.* *capsulatum*) | B39.3 | HIV | B20.1 | *E. coli*, CMV, *T. gondii* | B58.9 | Perfect |
| 25 | 32 | M | Disseminated infection (*H. capsulatum*) | B39.3 | HIV | B20 | *T. gondii*, CMV | B58.9 | Disseminated infection (*H. capsulatum*) | B39.3 | HIV | B20 | *T. gondii*, CMV | B58.9 | Perfect |
| 28 | 27 | M | Disseminated infection (*H. capsulatum*) | B39.3 | HIV | B20 |  |  | Disseminated infection (*H. capsulatum*) | B39.3 | HIV | B20 | *T. gondii* | B58.9 | Perfect |
| 42 | 78 | M | Disseminated infection (*H. capsulatum*) | B39.3 | HIV | B20 | *P. jirovecii, T. gondii, M. tuberculosis* | A19 | Disseminated infection (*H. capsulatum*) | B39.3 | HIV | B20 | *P. jirovecii, T. gondii, M. tuberculosis* | A19 | Perfect |
| 52 | 27 | F | Disseminated infection (*H. capsulatum*) | B39.3 | HIV | B20 | *T. gondii* | B58.9 | Disseminated infection (*H. capsulatum*) | B39.3 | HIV | B20 | *T. gondii* | B58.9 | Perfect |
| 57 | 34 | M | Disseminated infection (*H. capsulatum*) | B39.3 | HIV | B20 | *P. jirovecii* | B59 | Disseminated infection (*H. capsulatum*) | B39.3 | HIV | B20 | *P. jirovecii* | B59 | Perfect |
| ***Cryptococcus neoformans*** | | | |  |  |  |  |  |  |  |  |  |  |  |  |
| 8 | 28 | M | Disseminated infection (*C. neoformans*) | B45.7 | HIV | B20 | *M. tuberculosis* | A15 | Disseminated infection (*C. neoformans*) | B45.7 | HIV | B20 | *M. tuberculosis, T. gondii* | A15 | Perfect |
| 12 | 32 | M | Disseminated infection (*C. neoformans*) | B45.7 | HIV | B20 | *K. pneumoniae* | J15.6 | Disseminated infection (*C. neoformans*) | B45.7 | HIV | B20 |  |  | Perfect |
| 31 | 43 | M | Disseminated infection (*C. neoformans*) | B45.7 | HIV | B20 | *S. pneumoniae* | A40.3 | Disseminated infection (*C. neoformans*) | B45.7 | HIV | B20 | *S. pneumoniae* | A40.3 | Perfect |
| **Toxoplasma gondii** | | | |  |  |  |  |  |  |  |  |  |  |  |  |
| 4 | 38 | M | Disseminated infection (*T. gondii)* | B58.9 | HIV | B20 | CMV | B25.9 | Disseminated infection (*T. gondii)* | B58.9 | HIV | B20 | CMV | B25.9 | Perfect |
| 26 | 34 | M | Disseminated infection (*T. gondii)* | B58.9 | HIV | B20 | CMV | B25.9 | Disseminated infection (*T. gondii)* | B58.9 | HIV | B20 | CMV | B25.9 | Perfect |
| 38 | 46 | F | Disseminated infection (*T. gondii)* | B58.9 | HIV | B20 | *P. aeruginosa* | J15.1 | Meningoencephalitis  (*T. gondii)* | B58.2 | HIV | B20 | *P. aeruginosa* | J15.1 | Almost perfect |
| **Bacterial sepsis** | | | |  |  |  |  |  |  |  |  |  |  |  |  |
| 2 | 18 | F | Sepsis  (*N. meningitidis*) | A39.9 |  |  | *M. tuberculosis* | A15 | Sepsis  (*N. meningitidis*) | A39.9 |  |  |  |  | Perfect |
| 5 | 38 | M | Sepsis  (*N. meningitidis*) | A39.9 |  | A39.0 | Adenovirus | J12.0 | Sepsis  (*N. meningitidis*) | A39.9 |  |  |  |  | Perfect |
| 30 | 17 | F | Sepsis  (N. meningitidis) | A39.9 |  | A39.0 |  |  | Sepsis  (*N. meningitidis*) | A39.9 |  |  |  |  | Perfect |
| 13 | 18 | M | Sepsis  (*S. pneumoniae*) | A40.3 |  |  | Dengue virus | A90 | Sepsis  (*S. pneumoniae*) | A40.3 |  |  |  |  | Perfect |
| 51 | 31 | M | Sepsis  (*S. pneumoniae*) | A40.3 | HIV | B20 | *T. gondii* | B58.9 | Sepsis  (*S. pneumoniae*) | A40.3 | HIV | B20 |  |  | Perfect |
| **Disseminated viral diseases** | | | |  |  |  |  |  |  |  |  |  |  |  |  |
| 27 | 22 | F | Disseminated infection  (CMV) | B25.9 | HIV | B20 | *P. jirovecii, T. gondii* | B59 | Disseminated infection  (CMV) | B25.9 | HIV | B20 | *P. jirovecii* | B59 | Perfect |
| 59 | 30 | M | Disseminated infection  (CMV) | B25.9 | HIV | B20 | *M. tuberculosis* | A15 | Disseminated infection  (CMV) | B25.9 | HIV | B20 | *M. tuberculosis* | A15 | Perfect |
|  |  |  | **Other infections** |  |  |  |  |  |  |  |  |  |  |  |  |
| 32 | 13 | F | Disseminated infection (*Leishmania donovani*) | B55.0 |  |  |  |  | Disseminated infection (*Leishmania donovani*) | B55.0 |  |  |  |  | Perfect |
| 18 | 31 | M | Suggestive of viral hemorrhagic disease |  | HIV | B20 | *S. pneumoniae* | A40.3 | Suggestive of viral hemorrhagic disease |  | HIV | B20 | *S. pneumoniae, T. gondii* | A40.3 | Perfect |
| **Pulmonary infections** | | | |  |  |  |  |  |  |  |  |  |  |  |  |
| 36 | 35 | F | Pulmonary tuberculosis | A15 | HIV | B20 |  |  | Pulmonary tuberculosis | A15 | HIV | B20 |  |  | Perfect |
| 55 | 13 | M | Pneumonia  (*P. aeruginosa*) | J15.1 | Lupus Glomerulonephritis | N08.5 |  | M35.9 | Pneumonia  (*P. aeruginosa*) | J15.1 |  |  |  |  | Perfect |
| 56 | 39 | M | Pneumonia  (*P. jirovecii*) | B59 | HIV | B20 | CMV | B25.0 | Pneumonia  (*P. jirovecii*) | B59 | HIV | B20 | CMV | B25.0 | Perfect |
| 34 | 43 | M | Pneumonia  (CMV) | B25.0 | HIV | B20 |  |  | Pneumonia  (CMV) | B25.0 | HIV | B20 |  |  | Perfect |
| 61 | 50 | M | Pneumonia  (CMV) | B25.0 | HIV | B20 | *K. pneumoniae* | J15.6 | Pneumonia  (CMV) | B25.0 | HIV | B20 |  |  | Perfect |
| 14 | 72 | F | Pneumonia  (No agent) | J18 | Cirrhosis | K74.6 | Panhypopituitarism | E23.0 | Pneumonia  (No agent) | J18 | Cirrhosis | K74.6 |  |  | Moderate |
| 33 | 52 | M | Pneumonia  (No agent) | J18 | HIV | B20 | Meningoencephalitis | G04 | Pneumonia  (No agent) | J18 | HIV | B20 | Meningoencephalitis | G04 | Perfect |
| 45 | 33 | M | Organizing pneumonia secondary to infectious process | J84.8 | HIV | B21 |  |  | Organizing pneumonia secondary to infectious process | J84.8 | HIV | B21 |  |  | Perfect |
| **Central nervous system infections** | | | |  |  |  |  |  |  |  |  |  |  |  |  |
| 41 | 36 | F | Cerebral infection  (*T. gondii*) | B58.2 | HIV | B20 | CMV | B25.9 | Cerebral infection  (*T. gondii*) | B58.2 | HIV | B20 | CMV | B25.9 | Perfect |
| 44 | 30 | F | Cerebral infection  (*T. gondii*) | B58.2 | HIV | B22 | *K. pneumoniae* | J15.6 | Suggestive of cardiovascular disease | I51.6 | HIV | B22 |  |  | Discrepant |
| 46 | 46 | M | Cerebral infection  (*T. gondii*) | B58.2 | HIV | B22 | Hypertension | I10 | Suggestive of cardiovascular disease | I51.6 | HIV | B22 | *T. gondii* | B58.9 | Discrepant |
| 53 | 38 | M | Meningitis  (*C. neoformans*) | B45.7 | HIV | B20 | CMV | B25.0 | Pneumonia  (CMV) | B25.0 | HIV | B20 |  |  | Low |
| 60 | 34 | M | Meningitis  (*C. neoformans*) | B45.1 | HIV | B20 | *Histoplasma spp.,*  *T. gondii* | B39.2 | Pneumonia (*Histoplasma spp*.) | B39.2 | HIV | B20 | *T. gondii,* CMV*, P. jirovecii* | B25.0 | Low |
| 50 | 47 | M | Meningitis  (*N. meningitidis*) | A39.0 | HTLV I-II | Z22.6 | *S. pneumoniae* | A40.3 | Meningitis  (*N. meningitidis*) | A39.0 | HTLV I-II | Z22.6 | *S. pneumoniae* | A40.3 | Perfect |
| 54 | 69 | M | Meningitis  (*K. pneumoniae*) | G00.9 |  |  |  |  | Suggestive of cardiovascular disease | I51.6 |  |  |  |  | Discrepant |
| **Liver infections** | | | |  |  |  |  |  |  |  |  |  |  |  |  |
| 39 | 33 | M | Viral hepatitis  (HAV) | B15 |  |  |  |  | Viral hepatitis  (HAV) | B15 |  |  | Pneumonia  (No agent) |  | Perfect |
| **MALIGNANT TUMORS** | | | |  |  |  |  |  |  |  |  |  |  |  |  |
| 10 | 20 | M | Hepatocellular carcinoma (HBV) | C22.0 | Viral hepatitis (HBV+HDV) | B18.1 | Cirrhosis | K76.4 | Hepatocellular carcinoma (HBV) | C22.0 | Viral hepatitis (HBV+HDV) | B18.1 | Cirrhosis | K76.4 | Perfect |
| 22 | 48 | M | Hepatocellular carcinoma | C22.0 | Cirrhosis | K74.6 | *S. pneumoniae* | A40.3 | Hepatocellular carcinoma | C22.0 | Cirrhosis |  | *S. pneumoniae* | A40.3 | Perfect |
| 40 | 52 | M | Hepatocellular carcinoma (HBV) | C22.0 | Viral hepatitis  (HBV) | B18.1 | Cirrhosis | K74.6 | Hepatocellular carcinoma (HBV) | C22.0 | Viral hepatitis (HBV) | B18.1 | Cirrhosis, *E. coli* | K74.6 | Perfect |
| 47 | 34 | M | Hepatocellular carcinoma (HBV) | C22.0 | Viral hepatitis  (HBV) | B18.1 | Cirrhosis | K76.4 | Hepatocellular carcinoma (HBV) | C22.0 | Viral hepatitis (HBV) | B18.1 | Cirrhosis | K76.4 | Perfect |
| 9 | 37 | F* | Hepatocellular carcinoma (HBV) | C22.0 | Viral hepatitis (HBV)+HTLVI-II | B18.1 | Pneumonia  (no agent) | J18 | Pneumonia  (no agent) | J18 | Viral hepatitis (HBV)+HTLVI-II | B18.1 |  |  | Discrepant |
| 17 | 17 | F | Large B cell lymphoma | C83.3 | HIV | B20 | Pneumonia  (*M. tuberculosis)* | A19 | Pneumonia  *(M. tuberculosis)* | A15 | HIV | B20 |  |  | Discrepant |
| 19 |  |  | Disseminated Kaposi's sarcoma | C46 | HIV | B20 | Pneumonia | J18 | Disseminated Kaposi's sarcoma | C46 | HIV | B20 |  |  | Perfect |
| 20 | 39 | F | Disseminated breast ductal carcinoma | C50 | HIV | B21 |  |  | Disseminated breast ductal carcinoma | C50 | HIV | B21 | *C. krusei* | B37.9 | Perfect |
| 21 | 55 | F | Disseminated Adenocarcinoma of extrahepatic bile duct/pancreas | C24.0 | HIV | B20 |  |  | Cholangiocarcinoma | C22.1 | HIV | B20 |  |  | Moderate |
| 37 | 41 | F | Cholangiocarcinoma | C22.1 | Viral hepatitis (HBV+HDV) | B18.1 | Cirrhosis | K76.4 | Cholangiocarcinoma | C22.1 | Viral hepatitis (HBV+HDV) | B18.1 | Cirrhosis | K74.6 | Perfect |
| 43 | 81 | F | Disseminated gastric adenocarcinoma | C16 |  |  |  |  | Sepsis  *(P. aeruginosa*) | A41.5 |  |  |  |  | Discrepant |
| **OTHER DISEASES** | | | |  |  |  |  |  |  |  |  |  |  |  |  |
| 7 | 17 | F* | Pulmonary embolism secondary to sickle cell anemia | D57.0 | Maternal anemia complicating pregnancy | O99.0 | Pregnant | Z33 | Pulmonary infarct | I26 | Circulatory system disease complicating pregnancy | O99.4 | Pregnant | Z33 | None |
| 11 | 31 | M | Heart failure secondary to valvulopathy | I11.0 |  |  |  |  | Suggestive of cardiovascular disease. | I51.6 |  |  |  |  | Low |
| 15 | 18 | F | Liver necrosis secondary to chronic hepatitis | K73 | Viral hepatitis  (HBV) | B18.1 |  |  | Liver necrosis secondary to chronic hepatitis | K73 | Viral hepatitis (HBV) | B18.1 | *M. tuberculosis* | A15 | Perfect |
| 29 | 39 | M | Liver failure secondary to cirrhosis | K72.9 | Viral hepatitis (HBV+HDV) | B18.1 | Cirrhosis | K74.6 | Liver failure secondary to cirrhosis | K72.9 | Viral hepatitis (HBV+HDV) | B18.1 | Cirrhosis | K74.6 | Perfect |
| 48 | 25 | F | Liver necrosis and hemorrhage | K73 | Cirrhosis | K74.6 |  |  | Liver necrosis | K73 | Cirrhosis | K74.6 |  |  | Perfect |
| 49 | 67 | F | Non-alcoholic steatohepatitis | K75.8 | Cirrhosis | K74.6 | Thyroid hyperplasia | E04.9 | Steatohepatitis | K75.8 | Cirrhosis | K74.6 |  |  | Perfect |

* Maternal deaths: pregnant (case 7) and puerperal period (case 9)
